# Risk of temporal lobe necrosis between proton beam and volumetric modulated arc therapies in patients with different head and neck cancers

**DOI:** 10.1186/s13014-023-02344-y

**Published:** 2023-09-21

**Authors:** Chi-Hung Liu, Chien-Yu Lin, Bing-Shen Huang, Yi-Chia Wei, Ting-Yu Chang, Chih-Hua Yeh, Pi-Shan Sung, Jian-Lin Jiang, Li-Ying Lin, Joseph Tung-Chieh Chang, Kang-Hsing Fan

**Affiliations:** 1 Stroke Center, Department of Neurology, Linkou Medical Center, Chang Gung Memorial Hospital, Taoyüan, Taiwan; 2 School of Medicine, College of Medicine, Chang Gung University, Taoyüan, Taiwan; 3 Institute of Health Policy and Management, College of Public Health, National Taiwan University, Taipei, Taiwan; 4 Department of Radiation Oncology, Proton and Radiation Therapy Center, Chang Gung Medical Foundation, Linkou Chang Gung Memorial Hospital, Taoyüan, Taiwan; 5 Taipei Chang Gung Head and Neck Oncology Group, Chang Gung Memorial Hospital Linkou Medical Center, Taoyüan, Taiwan; 6 Particle Physics and Beam Delivery Core Laboratory of Institute for Radiological Research, Linkou Medical Center, Chang Gung University/Chang Gung Memorial Hospital, Taoyüan, Taiwan; 7 Department of Neurology, Keelung Chang Gung Memorial Hospital, Keelung, Taiwan; 8 Community Medicine Research Center, Keelung Chang Gung Memorial Hospital, Keelung, Taiwan; 9 Department of Neuroradiology, Linkou Medical Center, Chang Gung Memorial Hospital, Taoyüan, Taiwan; 10 Department of Neurology, College of Medicine, National Cheng Kung University Hospital, National Cheng Kung University, Tainan, Taiwan; 11 Department of Radiation Oncology, New Taipei Municipal Tu-Cheng Hospital, New Taipei City, Taiwan

**Keywords:** Temporal lobe necrosis, Head and neck cancer, Nasopharyngeal carcinoma, Radiation therapy, Proton therapy

## Abstract

**Background:**

To investigate the frequency of temporal lobe necrosis (TLN) soon after radiotherapy (RT) and identify differences among patients with various types of head and neck cancer (HNC) and between different RT methods.

**Methods:**

We retrospectively reviewed 483 patients with HNC who had completed RT in our hospital after January, 2015. These patients were followed-up at the radio-oncology department and received contrast-enhanced magnetic resonance imaging (MRI) or computed tomography (CT) to identify metastases or recurrence of cancer at regular intervals. Meanwhile, the occurrence of TLN, graded according to the Common Terminology Criteria for Adverse Events V5.0, was recorded. We categorized the patients into nasopharyngeal carcinoma (NPC) and non-NPC groups and compared the cumulative occurrence of TLN between the groups using Kaplan–Meier and Cox regression analyses. We further compared the cumulative occurrence of TLN between proton beam therapy (PBT) and volumetric modulated arc therapy (VMAT) in patients with any HNC, NPC, and non-NPC HNC.

**Results:**

Compared with the non-NPC group, the NPC group had a higher frequency of TLN (5.6% vs. 0.4%,  *p* < 0.01) and were more commonly associated with TLN in the Kaplan–Meier analysis (*p* < 0.01) and the Cox regression model after covariates were adjusted for (adjusted hazard ratio: 13.35, 95% confidence interval: 1.37–130.61) during the follow-up period. Furthermore, the frequency of TLN was similar between patients receiving PBT and those receiving VMAT (PBT vs. VMAT: 4.7% vs. 6.3%, *p* = 0.76). Kaplan–Meier analysis revealed that the accumulated risks of TLN were similar between PBT and VMAT in patients with any HNC (*p* = 0.44), NPC (*p* = 0.84), and non-NPC HNC (*p* = 0.70).

**Conclusion:**

Our study demonstrated that patients with NPC are susceptible to TLN during the early period after RT. In addition, PBT may be associated with an equivalent risk of TLN when compared with VMAT in patients with NPC or other HNCs.

**Supplementary Information:**

The online version contains supplementary material available at 10.1186/s13014-023-02344-y.

## Introduction

Radiotherapy (RT) is the standard treatment for head and neck cancer (HNC). Late effects on “bystander” organs have become increasingly prevalent in survivors of HNC. Among radiation injuries with long-term consequences, radiation vasculopathy and hypothyroidism are relatively common [1–3]. Patients treated with radical RT for HNC may receive significant radiation doses to large volumes of brain tissue. Patients with HNC, particularly advanced-stage nasopharyngeal carcinoma (NPC) may be at increased risk of adverse late brain effects including temporal lobe necrosis (TLN) after concurrent chemoradiation therapy [4, 5]. The mechanisms of TLN include microvascular injury, cell injury, and inflammatory and free radical injury [6]. Patients with TLN may develop epilepsy or cognitive decline. However, available data regarding the long-term prognosis of TLN remain scarce.

The application of proton beam therapy (PBT) in the treatment of HNC has been growing in the past few years [7]. The physical properties of the Bragg peak allow for precise dose delivery, thus minimizing or preventing an exit dose from affecting normal tissues located beyond the target. PBT is much more sensitive to tissue density than photon therapy. So far, intensity-modulated radiation therapy (IMRT) and PBT have been demonstrated to have similar treatment effects on local tumor control in patients with NPC [8, 9]. The accuracy achieved with PBT allows further widening of the therapeutic window, as dose escalation for radioresistant tumors is possible without jeopardizing treatment tolerance [10]. Comparative studies of PBT versus photon radiotherapy have demonstrated diverse results [11–15]. Some studies showed a lower regional toxicity in patients receiving PBT for various cancers [8, 9, 12, 13, 16], results corroborated by the biggest prospective PBT series published to date [12, 13]. However, other studies focusing on HNC only demonstrated that the risk of TLN was higher in patients receiving PBT [14, 15], which could be resulted from various biological and treatment uncertainties [16]. Studies mentioned above used different techniques treating different cancer around the brain, which were unable to answer the question. We used a PBT system with intensity modulation in our institution [17]. Intensity modulated proton therapy have been reported to improve several aspects of dose profile [18], and has become the preferred technique for HNC The current study focus on HNC treated by PBT with intensity modulation. Our work investigated the frequency of TLN during the early period after RT for HNC and the association between novel PBT and the risk of TLN in these patients.

## Materials and methods

### Patient recruitment and demographic data

**Fig. 1:**
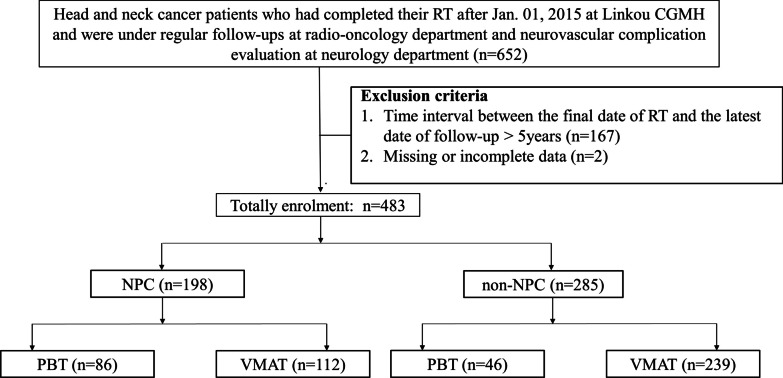
Patient enrollment. * CGMH* Chang Gung Memorial hospital; *HNC* Head and neck cancer; *NPC* Nasopharyngeal carcinoma; *PBT* Proton beam therapy; *RT* Radiotherapy; *VMAT* Volumetric modulated arc therapy

We reviewed patients with HNC who received RT after January 1, 2015, which was the date on which PBT was established in our institution. All enrolled patients received regular follow-ups with brain imaging studies to detect the presence of tumor recurrence at the radio-oncology department of our hospital. Patients were referred to the neurology department for evaluation of the risk of radiation vasculopathy or other neurovascular complications. We focused on TLN during the early period after RT. The median latency for TLN detection was 30 months with a range between 6 and 56 months after RT in previous study [19]; therefore, patients with a time interval of > 5 years between the date of their final RT and the date of their latest follow-up were excluded in this study. Data on demographic characteristics and RT of all recruited patients were recorded. Laboratory data, such as glycated hemoglobin, high and low-density lipoprotein cholesterol, and high-sensitivity C reactive protein (hs-CRP) levels, were registered (Fig. 1). The study was approved by the Ethics Institutional Review Board of our hospital (202101981B0 and 202200464B0).

### Cancer and RT data

We recorded the pathological types, locations, and tumor stages of HNC, method of RT [volumetric modulated arc therapy (VMAT) or PBT], interval from latest RT to study enrollment, and accumulated total doses of RT of each patient. Both PBT and VMAT treatment plans were generated using the Eclipse planning system (version 13.7; Varian Medical Systems, Palo Alto, CA, USA). Pencil beam scanning with 3 beam angles was used for full-field PBT plans [9]. The planning target volume in VMAT was expansion of 3–5 mm around the clinical target. For PBT, worst-case robust optimization was used for CTV coverage without PTV expansion. Dose constraints and algorithm used for optimization in the PBT were the same as those used in VMAT. The relative biological equivalent value of 1.1 was assumed for protons and calculated during optimization [20]. Prescription consisted of 6000–6600 centi-grays (cGy) 30–33 fractions for postoperative radiotherapy and 6996 cGy in 33 fractions for primary radiotherapy over 6–7 weeks (5 fractions per week). All targets were treated simultaneously [21].

### Grouping

Patients were categorized into two groups according to cancer pathology: an NPC group and a non-NPC group. The non-NPC group consisted of patients with oral cavity, oropharyngeal, laryngeal, and hypopharyngeal cancers. The NPC and non-NPC groups were further subdivided into PBT and VMAT subgroups based on the method of RT (Fig. 1).

### Follow-ups

Patients received regular follow-up at the radio-oncology department at least every 6 months and received at least one comprehensive neurological examination at the neurology department. Patients received contrast-enhanced magnetic resonance imaging (MRI) and/or computed tomography (CT) every 6 months to identify potential distal metastasis or recurrence of the primary tumor and to record the occurrence, type, and progression of TLN. CT of the head and neck region was performed using a multidetector CT scanner. Thin-slice CT images (3 mm in thickness) were reconstructed in the axial, coronal, and sagittal directions. MRI was performed using a 1.5 or 3.0 Tesla MR scanner by using a standard head and neck coil. Pre-contrast T1-weighted images, T2-weighted fat-saturated images, and postcontrast T1-weighted fat-saturated images were acquired in the axial, coronal, and sagittal planes. The section thicknesses were 5 mm with a 2.5-mm intersection gap in the axial plane and 4 mm with a 1-mm gap in the sagittal and coronal planes. Screening for TLN-associated neurological symptoms and detailed neurological examinations were performed during outpatient visits to the neurology department.

### Outcomes

The primary outcome in this study was the diagnosis of TLN. TLN was classified according to the pattern into three types: edema, enhancement, and necrosis [22]. The severity of TLN was graded according to the Common Terminology Criteria for Adverse Events (CTCAE) V5.0 [23].

### Radiation dose to the temporal lobe and dosimetry analysis

For patients who had TLN and were treated by photons radiotherapy (XRT) with single VMAT plan, dose statistics of bilateral temporal lobes were retrieved from VMAT plan used for treatment. In other patients with TLN, either treated by photons or protons, had adaptive replanning during treatment course to adaptive anatomical change during fractionated radiotherapy. The primary plans were registered to second plan with dose deformation and summation by VelocityTM oncology imaging informatics system (VARIAN, Palo Alto). We compared the dosimetry data among NPC patients receiving different method of RT and NPC patients with/without TLN. We also compared the dosimetry data of the diseased versus non-diseased lobes in NPC patients with TLN. Since only a small part of temporal lobe would be irradiated by PBT, maximal dose (Dmax) and highest dose delivered to small specific volume (0.5, 1, and 2 c.c.) should be more comparable. Shroeder and colleagues reported that D1cc is the most important dosimetry factor correlated to TLN [15]. The QUANTEC analysis also showed that maximal dose is predictive for TLN [24]. So Dmax, D0.5 cc, D1cc, and D2cc were reported and analyzed. On the other hand, spread of low and intermediate radiation dose is the main disadvantage of VMAT. Therefore, the volumes of temporal lobe that exposed to radiation doses of 2000 (V20Gy) and 5000 cGy (V50Gy) or more were also analyzed [25–27].

### Statistical analysis

We used SPSS 22.0 (SPSS, Chicago, IL, USA) to analyze the clinical data. Parameters are presented as mean ± standard deviation or frequency (%). We used an independent two-sample *t* test to identify differences in the continuous variables between the study groups. Categorical variables were compared using a chi-square test or Fisher’s exact test. Radiation dose and follow-up duration were analyzed using Mann-Whitney tests and were presented as median (quartile 1, quartile 3). Intergroup differences in event risk and time to TLN were compared using Kaplan–Meier analysis and the log-rank test. Cox regression analysis was used to compare risks between the study groups, in which covariates [age, tumor staging (T3 or T4), cancer type (NPC or non-NPC), RT doses, time interval to the end of RT, type of RT (PBT or VMAT), and reirradiation) were adjusted. Continuous variables (RT dose) were analyzed as continuous data instead of being categorized into groups. We used a univariate Cox regression model and a multivariable Cox regression model with backward selection to investigate the correlations between these risk factors, NPC, and the risk of TLN. Since the definition of tumor stage is different between NPC and non-NPC, which could extra-ordinary be a potential confounding factor to the study results. Cochran–Mantel–Haenszel test was used to assess the conditional independence of cancer types associated with TLN. Significance was indicated by *p* < 0.05.

## Results

A total of 652 patients with HNC who finished RT after January 1, 2015, received regular post-RT follow-ups, and had evaluation for neurovascular complications screening by neurologists in our hospital were initially reviewed. Of these patients, 167 whose time interval between the date of their final RT and the date of their latest follow-up was > 5 years were excluded. Two patients with missing information were also excluded. In total, 483 patients with HNC were recruited and categorized into two groups: 198 (41.0%) were in the NPC group and 285 (59.0%) were in the non-NPC group. In the NPC group, 86 (43.4%) patients received PBT and 112 (56.6%) patients received VMAT. In the non-NPC group, 46 (16.1%) patients received PBT and 239 (83.9%) patients received VMAT (Fig. 1).

Compared with the patients in the NPC group, those in the non-NPC group were older (non-NPC vs. NPC: 58.30 ± 9.89 vs. 50.17 ± 10.68 years, *p* < 0.01) and were more likely to be men (non-NPC vs. NPC: 90.9% vs. 81.3%, *p* < 0.01), had higher frequencies of betel quid chewing (non-NPC vs. NPC: 43.4% vs. 14.2%, *p* < 0.01) and smoking (non-NPC vs. NPC: 57.2% vs. 38.3%, *p* < 0.01). The frequencies of stage T4 cancer (NPC vs. non-NPC: 29.7% vs. 35.6%, *p* = 0.18) and advanced cancer (stages T3 and T4; NPC vs. non-NPC: 55.9% vs. 60.1%, *p* = 0.37) were similar between the two groups. Patients in the NPC group were more likely to receive PBT (NPC vs. non-NPC: 43.4% vs. 14.2%, *p* < 0.01) and had higher mean RT doses [NPC vs. non-NPC: 6996 (6996, 6996) vs. 6600 (6600, 6996) cGy, *p* < 0.01]. Levels of metabolic parameters, namely glycated hemoglobin, free T4 levels, and inflammatory markers, including hs-CRP and homocysteine, were similar between the two groups (Table 1).

**Table 1:** Baseline characteristics of the study groups

	NPC (*n* = 198)	Non-NPC (*n* = 285)	*p* value
Demographics			
Age (years)	50.17 ± 10.68	58.30 ± 9.89	< 0.01^†^
Sex (% male)	161 (81.3%)	259(90.9%)	< 0.01^†^
Hypertension (%)	29 (14.6%)	58 (20.5%)	0.16
Diabetes mellitus (%)	18 (9.1%)	35 (12.4%)	0.42
Dyslipidemia (%)	35 (17.7%)	45 (15.9%)	0.61
T4 stage (%)	58 (29.7%)	99 (35.6%)	0.18
Advanced T (T3/T4) stages (%)	109 (55.9%)	167 (60.1%)	0.37
Smoking (%)	76 (38.3%)	162 (57.2%)	< 0.01^†^
Betel quid chewing (%)	28 (14.2%)	123 (43.4%)	< 0.01^†^
RT dose (centi-grays)	6996 (6996, 6996)	6600 (6600, 6996)	< 0.01^†#^
Proton beam therapy (%)	86 (43.4%)	46 (16.1%)	< 0.01^†^
Re-irradiation	11 (5.6%)	19 (6.7%)	0.63
Cancer types			–
NPC	198 (100%)	0 (0%)	
Oral cavity/oropharyngeal cancer	0 (0%)	199 (69.8%)	
Laryngeal cancer	0 (0%)	23 (8.1%)	
Hypopharyngeal cancer	0 (0%)	56 (19.6%)	
Others	0 (0%)	7 (2.5%)	
Laboratory data			
Glycated hemoglobin (%)	5.66 ± 0.54	6.31 ± 0.69	0.28
Creatinine (mg/dL)	1.02 ± 1.08	1.04 ± 1.04	0.91
Homocysteine	12.05 ± 5.90	12.69 ± 4.83	0.31
High-sensitivity CRP	3.25 ± 6.51	10.84 ± 54.50	0.07
Uric acid (mg/dL)	6.79 ± 9.39	7.60 ± 13.25	0.51
Triglyceride (mg/dL)	129.39 ± 93.59	147.03 ± 95.69	0.07
Free T4	1.25 ± 3.69	1.38 ± 6.78	0.83
Time from RT to the last follow-up (months)	35 (21, 46)	30 (18, 42)	0.03^†#^

*CRP* c-reactive protein;* NPC* Nasopharyngeal carcinoma;* RT* Radiotherapy

^†^*p* < 0.05

^#^Data were examined using Mann-Whitney exams and presented as median (quartile 1, quartile 3)

Other data were examined using two-sample *t* tests (continuous variables) and chi-square tests (categorical variables)

**Fig. 2:**
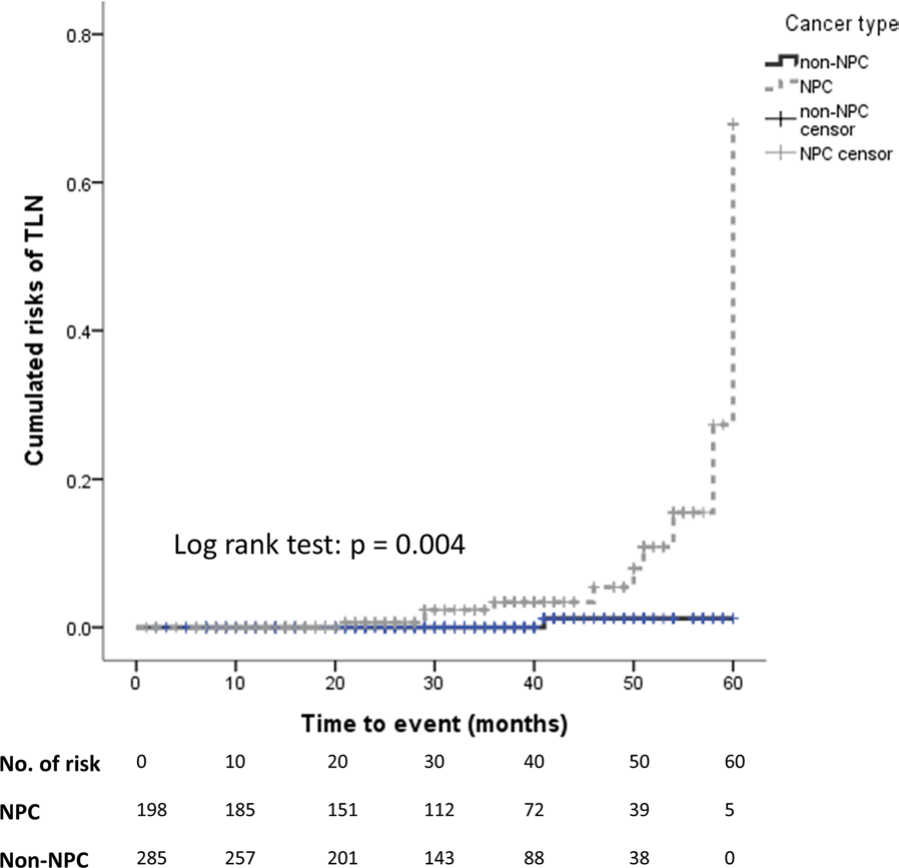
Cumulative risks of TLN in patients with NPC and non-NPC head and neck cancer. Kaplan–Meier analysis comparing the cumulative risks of TLN between the NPC and non-NPC groups. Frequency of TLN was significantly higher in the NPC group than in the non-NPC group. *NPC* Nasopharyngeal carcinoma; *TLN* Temporal lobe necrosis

The mean interval from RT completion to the last follow-up was slightly longer in the NPC group [NPC vs. non-NPC: 35 (21, 46) vs. 30 (18, 42) months, *p* = 0.03]. Compared with the non-NPC group, the patients in the NPC group were more likely to receive a diagnosis of TLN (NPC vs. non-NPC: 5.6% vs. 0.4%, *p* < 0.01; Table 2) and were at a higher risk of developing TLN during the follow-up according to the Kaplan–Meier analysis (log–rank test *p* < 0.01; Fig. 2) and the Cox regression model (adjusted hazard ratio: 13.35, 95% confidence interval: 1.37-130.61, *p* = 0.03) after adjustment for covariables. Although the definition of advanced T stage could be different between NPC and non-NPC, both the NPC patients in the T0-T2 stage (NPC vs. non-NPC: 4.7% vs. 0.0%, p = 0.04; Additional file 1: Table 1) and those in the T3-T4 stage (NPC vs. non-NPC: 6.4% vs. 0.6%, *p* < 0.01) yielded higher risks of TLN development. Cochran–Mantel–Haenszel test (*p* < 0.01) also showed that patients with NPC remained have a higher odds ratio of the TLN occurrence after excluding the effect of tumor stages.

**Table 2:** Primary study outcome and associated factors of TLN in Cox regression models

	NPC, n = 198 (%)	Non-NPC, n = 285 (%)	*p* value	
*Primary analysis*				
Occurrence of TLN	11 (5.6)	1 (0.4)		< 0.01^#^

	Adjusted HR (95% CI)	*p* value
*Multi-variable analysis*		
Cancer type (NPC vs. non-NPC)	13.35 (1.37, 130.61)	0.03^†^
Age	1.04 (0.98, 1.11)	0.16
T3/T4 stage	3.27 (0.80, 13.27)	0.10
Radiation doses	1.00 (1.00, 1.00)	0.01
Proton-beam therapy	1.67 (0.40, 6.94)	0.48
Re-irradiation	0.87 (0.04, 19.60)	0.93
Time to radiation therapy	1.00 (0.95, 1.05)	0.96

*TLN* Temporal lobe necrosis; *CI* Confidence interval; *HR* Hazard ratio; *NPC* Nasopharyngeal carcinoma

^#^ Data was analyzed using the Chi-square test

^†^ Data was analyzed using a multivariable Cox regression model with backward selection

**Fig. 3:**
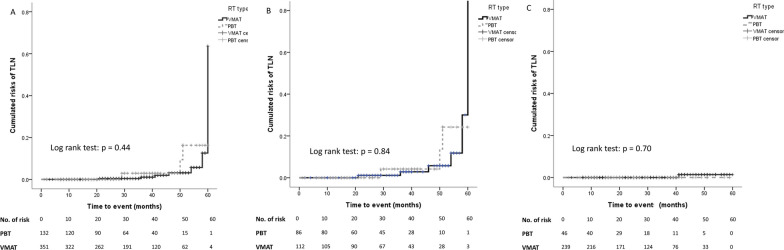
Cumulative risks of TLN of patients with any HNC, NPC, and non-NPC HNC receiving PBT or VMAT. Kaplan–Meier analysis comparing the cumulative risks of TLN between PBT and VMAT in patients with any HNC (**A**), patients with NPC (**B**), and patients with non-NPC HNC (**C**). The risks of TLN were comparable between PBT and VMAT in all the subgroups. * HNC* Head and neck cancer; *NPC* Nasopharyngeal carcinoma; *PBT* Proton beam therapy; *TLN* Temporal lobe necrosis; *VMAT* Volumetric modulated arc therapy

Regarding the influence of the method of RT, the frequency of TLN was similar between patients receiving PBT and VMAT (PBT vs. VMAT: 4.7% vs. 6.3%, *p* = 0.76). Kaplan–Meier analysis indicated that the accumulated risks of developing TLN between patients who received PBT and those who received VMAT were similar among patients with any HNC (log–rank test: *p* = 0.44; Fig. 3A), patients with NPC (log–rank test: *p* = 0.84; Fig. 3B), and patients with non-NPC HNC (log–rank test: *p* = 0.70; Fig. 3C). The radiation doses to the temporal lobes in the NPC patients receiving different method of RT were shown in the Table 3. Compared to NPC patients undergoing VMAT therapy, NPC patients receiving PBT had significantly lower V20Gy and V50Gy of the temporal lobes. D1cc and D2cc of left temporal lobe were also significantly lower in patients under PBT (Table 3).

**Table 3:** Comparisons of dosimetry data in NPC patients receiving PBT versus VMAT and with versus without TLN

	Radiation dose to right temporal lobe						Radiation dose to left temporal lobe						Staging distribution	
	Dmax	D0.5 cc	D1cc	D2cc	V50Gy (c.c.)	V20Gy (c.c.)	Dmax	D0.5 cc	D1cc	D2cc	V50Gy (c.c.)	V20Gy (c.c.)	T4 (%)	T3/T4 (%)
*VMAT versus PBT*														
VMAT	7297 (5994, 7460)	6678 (5619,7198)	6307 (5454,7009)	5906 (4836,6477)	4.38 (1.67,8.09)	26.41 (15.55,41.36)	7342 (5963,7493)	6445 (5615,7321)	6196 (5397,7248)	5855 (4832,7135)	4.64 (1.80,8.93)	24.00 (14.65,40.36)	34.90%	60.60%
PBT	6880 (6467,7414)	6057 (5472,6867)	5675 (5108,6627)	5076 (4485,6211)	2.15 (1.23,5.68)	11.89 (9.63,19.86)	6979 (6027,7379)	5980 (5340,6845)	5650 (4952,6548)	5001 (4303,6169)	2.03 (0.81,4.98)	12.55 (8.27,18.12)	23.30%	50.00%
p value	0.71	0.32	0.15	0.05	0.02	< 0.01	0.82	0.10	0.03	< 0.01	< 0.01	< 0.01	0.08	0.14
*With TLN versus without TLN*														
With TLN	7453 (7137,7489)	7070 (6345,7359)	6873 (6078,7305)	6489 (5854,7208)	7.00 (5.00,9.00)	43.00 (18.54,53.00)	7583 (6713,7722)	7300 (6426,7448)	7018 (6250,7409)	6651 (5849,7357)	8.00 (4.00,16.00)	40.00 (20.00,51.00)	45.50%	63.60%
W/O TLN	7096 (6283,7444)	6211 (5497,7082)	5870 (5221,6807)	5491 (4590,6320)	3.01 (1.39,6.62)	17.97 (10.56,27.65)	7115 (5983,7443)	6192 (5412,7148)	5806 (5100,6972)	5346 (4413,6486)	2.71 (1.14,6.54)	15.77 (10.31.26.56)	28.80%	55.40%
p value	0.03	0.01	< 0.01	< 0.01	< 0.01	0.03	0.01	< 0.01	< 0.01	< 0.01	< 0.01	< 0.01	0.31	0.6

The doses were presented as median (quartile 1, quartile 3). The data were examined using Mann–Whitney test

*Gy* Grays; *NPC* Nasopharyngeal carcinoma; *PBT* Proton beam therapy; *TLN*, Temporal lobe necrosis; *VMAT* Volumetric modulated arc therapy; *W/O* Without

 In total, 12 patients (2.5%) developed TLN. The details of the RT dose-volume parameters of the 12 patients who developed TLN are listed in Additional file 1: Table 2. Among these patients, 3 (25%) had bilateral involvement, 11 (91.7%) developed an edematous pattern, and 6 (50%) had contrast enhanced lesions. Only 1 (8.3%) patient had a seizure before the latest follow-up. Ten (83.3%) of the 12 TLN patients were grade 1 (asymptomatic) in severity based on CTCAE v5.0 criteria, while 2 (Patient 4 and 6, 16.7%; Additional file 1: Table 2) were graded 2 and received steroid treatment. The dose distributions of select patients with NPC with and without TLN receiving different RT methods are shown in the Supplementary Figure. Of notes, all dosimetry parameters (Dmax, D0.5 cc, D1cc, D2cc, V50Gy, and V20Gy) in bilateral temporal lobes of the NPC patients with TLN were higher compared to other NPC patients without TLN (Table 3). Totally 15 necrotic temporal lobes were observed in NPC patients with TLN, all the dosimetry parameters except V20Gy of the necrotic temporal lobes were higher than those of the non-necrotic temporal lobes with statistical significance (Additional file 1: Table 3).

## Discussion

Our study demonstrated that patients with NPC were more susceptible to TLN development during the early period after RT than patients with non-NPC HNC. In addition, PBT is not associated with an increased risk of TLN compared with VMAT in patients with any HNC, NPC, or non-NPC HNC. The incidence rate of TLN varied from 4.6 to 7% during the early period after RT [26]. NPC is known to be a risk factor for TLN; however, the prevalence of TLN in patients with non-NPC HNC remains uncertain. For patients with non-NPC HNCs, exposure of high radiation dose over temporal lobes is not rare. The treatment volume of RT would extend to skull base or temporal lobes if there is extensive perineural invasion in pathological samples or if patients are at risk of retropharyngeal lymph node metastasis. One previous study investigated the association of RT with the development of TLN in patients with parotid gland tumors [28]; One of our patients with parotid gland tumor also developed TLN during follow-up. However, our results revealed the risk of TLN is relatively lower in patients with non-NPC HNC. It is possible that the prescription dose to skull base for the conditions described above could be definitively lower than the radiation dose prescribed for the patients with overt tumor extension to skull base. Therefore, screening for TLN in non-NPC population may not be cost-effective.

For patients with NPC, an important question is whether the RT method moderates the risk of TLN, particularly in the current era, where several advanced RT methods are available. A study demonstrated that IMRT significantly reduced the risk of TLN compared with two-dimensional RT [29]. One study revealed that the prevalence rate of 2-year TLN was approximately 4.6% in patients with HNC after PBT [26]. Most of the studies exploring the cause of TLN showed that highest dose in a small area is more critical in the prediction of TLN than the total volumes of intermediate radiation dose. However, the most significant advantage of PBT could be the decreasing “dose spillage”, which means the intermediate and low dose region spreading everywhere in the body. This could be the reason why PBT may not reduce the risk of TLN in these studies [26]. However, another study demonstrated that the risk of developing TLN could be even higher in patients with NPC who received PBT (10%) than in those who received VMAT (4%), and this may arouse the attention to possible adverse effects of PBT use in NPC patients [11]. Compared with the patients undergoing VMAT treatment, our NPC patients who received PBT had similar risks of TLN development during follow-up, and had lower V20Gy and V50Gy radiation doses bilaterally. Our findings might mitigate some safety concerns that individuals may have regarded PBT as a risk factor for TLN in patients with NPC, and may provide some evidence of post-RT complication for the share-decision-making process before choosing the treatment strategy.

Advanced T staging, RT dose-volume parameters, radiation fraction, and reirradiation are common risk factors for TLN development [30]. However, there were still 4 NPC patients with T2 stage developed TLN in our study. In these patients, the extents of parapharyngeal space invasion of the primary tumor were close to the cranial cavity, and therefore the radio-oncologists in-charge extended the treatment volume into the cranial cavity and along the foramen ovale to eradicate possible subclinical tumor extension. The radiation doses to the lowest part of the temporal lobes were more than 7000 cGy in these patients, and this could be a possible reason why these NPC patients with T2 stage still developed TLN after RT. The current study showed that higher radiation doses to the temporal lobe increased the risk of TLN, suggesting the radiation dose is a more determinative factor rather than the method of radiation. However, this study was not able to identify the most decisive dose level for predicting TLN. Different methods of dose-response analysis would be required to solve this problem. To avoid TLN, we should still follow the QUANTEC report for dose prescription to reduce maximal dose to temporal lobe currently [25].

The median follow-up time was approximately 3.43 years in TLN patients after RT [31]. Among patients who developed TLN, 21.5% developed symptomatic epilepsy during follow-up [31]. Another retrospective review found that 88.5% of patients developed radiation necrosis after RT, of which 16.5% developed epilepsy [32]. However, the incidence rates of TLN and seizure in our study were lower than those in previous studies. The pathogenesis of RT-related epilepsy can be explained by various potential mechanisms: (1) RT can cause endothelial cell and blood–brain barrier damage and aggravate brain edema, and (2) inflammatory cytokines released after RT may be associated with epilepsy development [33]. Detection of epilepsy is important, because seizures can lead to status epilepticus, sudden unexplained death in epilepsy, conscious disturbance, cognitive impairment, and significant morbidity. Notably, temporal lobe epilepsy should theoretically be higher in patients after RT due to its anatomical correlation. Our patient with epilepsy developed an altered sense of smell, which was considered as grade 1 seizure based on CTCAE v5.0 [23]; this symptom and interictal discharges on electroencephalogram disappeared after treatment with levetiracetam. Seizure symptoms are sometimes vague in these TLN patients. Cooperation between different medical specialties may help to discover the occurrence of seizure. Cognitive impairment is another complication in patients with TLN [34–36]. Cerebral microbleeds, hippocampal atrophy, and TLN have been proposed as possible mechanisms leading to cognitive impairment after RT [34–36]; however, our retrospective study was unable to obtain comprehensive neuropsychiatric assessments in these patients. Future prospective studies may help to illustrate whether the method of RT affects cognitive or hippocampus preservation [37, 38].

A previous study showed that TLN usually begins with white matter lesions, followed by contrast-enhanced lesions and cystic formation. These lesions can be progressed, regressed, static, or fluctuated [39]. In our patients, 92% and 50% exhibited white matter and contrast-enhanced lesions, but none had cystic formation, which is consistent with previous reports. Treatment of TLN remains an area of uncertainty. Glucocorticoid, anticoagulant, pentoxifylline with vitamin E, bevacizumab, hyperbaric oxygen therapy, and surgical treatment have all been proposed for TLN treatment [40–42]. The efficacy of steroid treatment varies, and previous reports have shown that 19.4% and 15.3% of patients with TLN achieve complete response and partial response, respectively, after oral steroid treatment [30, 43]. However, steroid use is not a standard treatment of TLN in our hospital; thus, this study was unable to investigate the responses to the aforementioned treatments.

This study has several limitations. First, the sample size of enrolled patients may not be sufficient to yield results of clinical significance. Our hospital is a large medical center in East Asia providing PBT to patients with HNC and may provide valuable pilot data in this field. A multicenter prospective study is warranted to evaluate the accuracy of our findings [38]. Second, both contrast-enhanced CT and MRI studies were arranged to screen for cancer recurrence in these patients. MRI was more sensitive to TLN diagnosis than was CT. The retrospective nature of our study may have underestimated the true prevalence of TLN in patients with HNC. Third, dosimetry data were not available for all patients. This may have affected the determination of the causal relationship between TLN and the method of RT. However, the purpose of this study was to investigate the associations of TLN with different types of HNC and RT methods instead of determining causal relationships. Fourth, a wide range of confounding factors was present. Selection and reporting bias due to influencing factors and missing data may also have confounded the results of the present study. Finally, the generalizability of our results to patients of other ethnicities remains uncertain. In addition, the capacity of PBT may still be limited and therefore patient selection remains crucial. Also, access is not equitable in most countries and it needs to be clear that in a wide territory with a high incidence of NPC, patients can still be treated with gold-standard XRT without jeopardizing their outcomes [44, 45].

## Conclusions

Our single-center retrospective study demonstrated that patients with NPC were more susceptible to TLN development during the early period after RT than patients with non-NPC HNC. Among advanced RT techniques, PBT was associated with an equivalent risk of TLN compared with VMAT in patients with NPC. These findings could potentially mitigate the safety concerns regarding the use of PBT in these patients.

### Supplementary Information

**Additional file 1: Figure.** Radiation dose distributions of select patients with nasopharyngeal carcinoma receiving RT. **Table 1.** Risk of TLN and NPC, overall and by T stage category. **Table 2.** Characteristics of patients with TLN in this study. **Table 3.** Comparisons of dosimetry data of the TLN versus non-TLN lobes in NPC patients with TLN.

## Data Availability

The data sets generated during this study are available from the corresponding author upon reasonable request.
