# Supplementary material for: Risk of temporal lobe necrosis between proton beam and volumetric modulated arc therapies in patients with different head and neck cancers
Source: Radiat Oncol. 2023 Sep 21;18:155. doi: 10.1186/s13014-023-02344-y (PMC10512503; doi:10.1186/s13014-023-02344-y)
Supplement: Supplementary file 1 — Additional file 1: Figure. Radiation dose distributions of select patients with nasopharyngeal carcinoma receiving RT. Table 1. Risk of TLN and NPC, overall and by T stage category. Table 2. Characteristics of patients with TLN in this study. Table 3. Comparisons of dosimetry data of the TLN versus non-TLN lobes in NPC patients with TLN. [file 13014_2023_2344_MOESM1_ESM.pdf]

Supplementary materials

Supplementary Figure. Radiation dose distributions of select patients with nasopharyngeal carcinoma receiving RT.

Supplementary Figure A and B showing the radiation dose distributions in two selected patients who did not have TLN, while Supplementary Figure C and D showing the radiation dose distributions in the other two selected patients with TLN. The red-lines circles the area with contrast enhancement on T1 weighted images, and the purple lines circles the hyperintensity area on T2 weighted images in the Figure C and D. The selected patients in the Supplementary Figure A and C received VMAT, whereas those in the Supplementary Figure B and D received PBT.
PBT, proton beam therapy; TLN, temporal lobe necrosis; VMAT, volumetric modulated arc therapy.

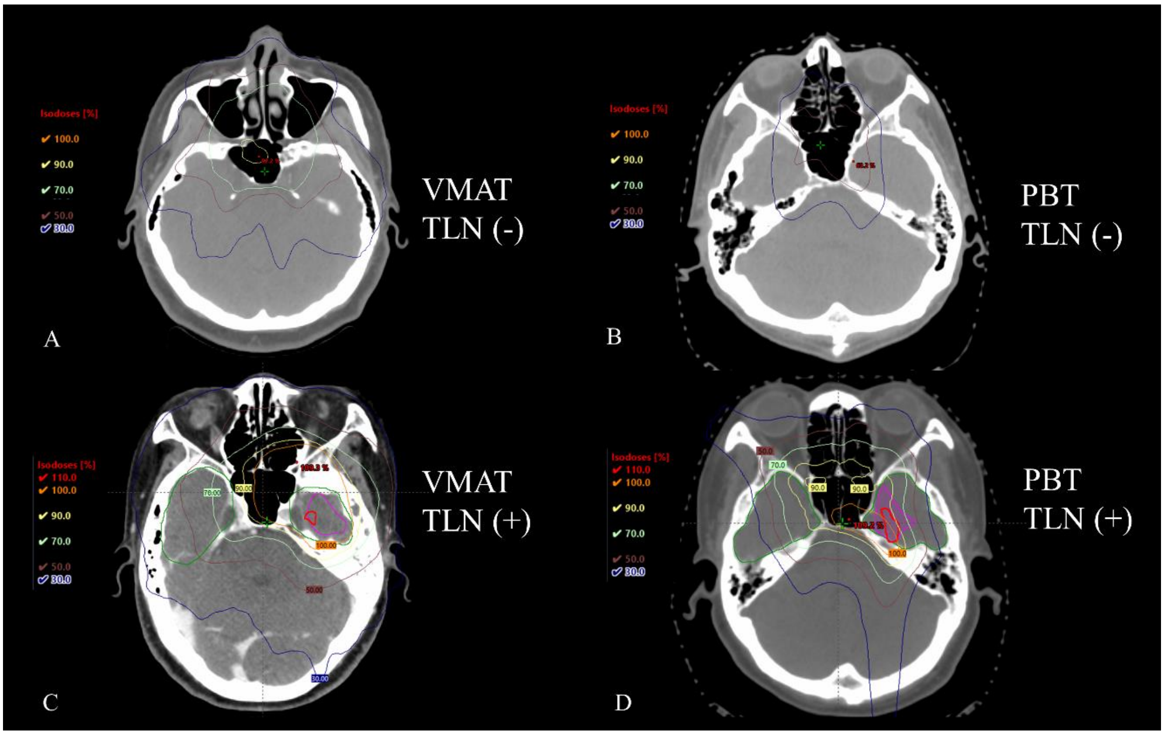

Supplementary Table 1. Risk of TLN and NPC, overall and by T stage category

	T stage					
	T0-T2		T3-T4		Total	
	NPC	non-NPC	NPC	non-NPC	NPC	non-NPC
TLN (n)	4	0	7	1	11	1
Total at risk (n)	86	111	109	165	195	276
Risk of TLN (%)	4.7%	0.0%	6.4%	0.6%	5.6%	0.4%
Odds ratio		NA		11.26 (1.37-92.82)		16.44 (2.11-128.42)
p value (X^2 test)		0.04		<0.01		<0.01

Mantel-Haenszel test, p<0.01

NPC, nasopharyngeal carcinoma; TLN, temporal lobe necrosis

Odds ratio was interpretated as ratio (95% confidence interval)

Supplementary Table 2. Characteristics of patients with TLN in this study

	Baseline characteristics				RT data			TLN type				SE	RT dose	Right temporal total						Left temporal total					
	**Age**	**Sex**	**Cancer**	**Staging**	**CCRT**	**re-RT**	**RT type**	**Edema**	**CE**	**NEC**	**Loc**		**(cGy)**	**Dmax**	**D0.5cc**	**D1cc**	**D2cc**	**V50Gy(c.c.)**	**V20Gy(c.c.)**	**Dmax**	**D0.5cc**	**D1cc**	**D2cc**	**V50Gy(c.c.)**	**V20Gy(c.c.)**
**1**	70	F	NPC	T3N1M0	+	-	PBT	+	+	-	Lt	-	6996	7488	6876	6610	6495	7	19	7583	7215	7019	6651	8	19
**2**	52	F	NPC	T4N1M0	+	-	PBT	+	-	-	Lt	-	7000	6611	5979	5582	4949	2	10	7722	7424	7359	7260	12	23
**3**	57	F	NPC	T2N1M0	+	-	VMAT	+	-	-	Bil	-	7000	7167	6716	6404	5629	3	8	6713	6317	6003	5147	2	9
**4**	56	M	NPC	T4N0M0	+	+	VMAT	+	+	-	Bil	+	6996	7453	7329	7305	7208	9	60	7892	7812	7717	7513	18	51
**5**	74	F	NPC	T4N1M0	+	-	VMAT	+	-	-	Lt	-	6996	7471	6345	6073	5925	6	48	7708	7573	7524	7461	23	50
**6**	45	M	NPC	T2N2M0	+	-	VMAT	+	-	-	Bil	-	6996	7543	7519	7394	7355	12	53	7513	7378	7299	6884	7	56
**7**	72	M	Parotid	T4N2M0	+	-	VMAT	-	+	-	Rt	-	6996	7168	6915	6831	6607	9	25	3379	2952	2770	2524	0	17
**8**	56	M	NPC	T3N2M0	+	-	VMAT	+	-	-	Lt	-	6996	7453	7070	6873	6443	7	43	7598	7448	7409	7357	16	52
**9**	53	F	NPC	T4N1M0	+	-	VMAT	+	+	-	Rt	-	6996	7489	7373	7351	7324	22	54	5883	5620	5550	5345	3	40
**10**	48	M	NPC	T2N1M0	+	-	PBT	+	+	-	Lt	-	6996	6686	6178	6078	5854	5	19	8132	7300	6994	6508	9	28
**11**	50	M	NPC	T2N3M0	+	-	VMAT	+	-	-	Rt	-	6996	7307	7124	6920	6489	8	43	6528	6426	6360	6143	8	50
**12**	49	F	NPC	T4N1M0	+	-	PBT	+	+	-	Rt	-	6996	7556	7359	7270	6956	7	22	6797	6427	6250	5849	4	20

Bil, bilateral TLN; CCRT, concurrent chemoradiation therapy; CE, contrast-enhanced lesion; cGy, centigray; F, female; Loc, TLN location; Lt, Lt TLN; M, male; NEC, necrosis; NPC, nasopharyngeal carcinoma; PBT, proton beam therapy; RT, radiotherapy; Rt, right TLN; SE, seizure; TLN, temporal lobe necrosis; VMAT, volumetric modulated arc therapy.

Supplementary Table 3. Comparisons of dosimetry data of the TLN vs. non-TLN lobes in NPC patients with TLN

	**Radiation dose to the TLN vs. non-TLN lobes in NPC patients with TLN**					
	**Dmax**	**D0.5cc**	**D1cc**	**D2cc**	**V50Gy (c.c.)**	**V20Gy (c.c.)**
TLN lobe	7550(7417,7712)	7386(7192,7466)	7302(6976,7398)	7082(6503,7356)	9.00(7.00,16.50)	46.50(21.29,53.25)
Non-TLN lobe	6742(6549,7467)	6366(6029,6764)	6164(5705,6548)	5890(5471,6368)	5.50(3.25,6.91)	30.00(18.65,46.75)
p value	<0.01	<0.01	<0.01	<0.01	0.01	0.17

Gy, Grays; NPC, nasopharyngeal carcinoma; TLN, temporal lobe necrosis; The doses were presented as median (quartile 1, quartile 3).

The data were examined using Mann-Whitney test
